# Effects of hyperaemia on left ventricular longitudinal strain in patients with suspected coronary artery disease: A first-pass stress perfusion cardiovascular magnetic resonance imaging study

**DOI:** 10.1007/s12471-017-1071-3

**Published:** 2018-01-08

**Authors:** P. Garg, R. Aziz, T. Al Musa, D. P. Ripley, P. Haaf, J. R. J. Foley, P. P. Swoboda, G. J. Fent, L. E. Dobson, J. P. Greenwood, S. Plein

**Affiliations:** Multidisciplinary Cardiovascular Research Centre & Division of Biomedical Imaging, Leeds Institute of Cardiovascular and Metabolic Medicine, University of Leeds, Leeds, UK

**Keywords:** Cardiovascular magnetic resonance, Coronary artery disease, Adenosine, Perfusion imaging, Left ventricular function

## Abstract

**Aims:**

Myocardial perfusion imaging during hyperaemic stress is commonly used to detect coronary artery disease. The aim of this study was to investigate the relationship between left ventricular global longitudinal strain (GLS), strain rate (GLSR), myocardial early (E’) and late diastolic velocities (A’) with adenosine stress first-pass perfusion cardiovascular magnetic resonance (CMR) imaging.

**Methods and results:**

44 patients met the inclusion criteria and underwent CMR imaging. The CMR imaging protocol included: rest/stress horizontal long-axis (HLA) cine, rest/stress first-pass adenosine perfusion and late gadolinium enhancement imaging. Rest and stress HLA cine CMR images were analysed using feature-tracking software for the assessment of myocardial deformation. The presence of perfusion defects was scored on a binomial scale. In patients with hyperaemia-induced perfusion defects, rest global longitudinal strain GLS (−16.9 ± 3.7 vs. −19.6 ± 3.4; *p*-value = 0.02), E’ (−86 ± 22 vs. −109 ± 38; *p*-value = 0.02), GLSR (69 ± 31 vs. 93 ± 38; *p*-value = 0.01) and stress GLS (−16.5 ± 4 vs. −21 ± 3.1; *p* < 0.001) were significantly reduced when compared with patients with no perfusion defects. Stress GLS was the strongest independent predictor of perfusion defects (odds ratio 1.43 95% confidence interval 1.14–1.78, *p*-value <0.001). A threshold of −19.8% for stress GLS demonstrated 78% sensitivity and 73% specificity for the presence of hyperaemia-induced perfusion defects.

**Conclusions:**

At peak myocardial hyperaemic stress, GLS is reduced in the presence of a perfusion defect in patients with suspected coronary artery disease. This reduction is most likely caused by reduced endocardial blood flow at maximal hyperaemia because of transmural redistribution of blood flow in the presence of significant coronary stenosis.

**Electronic supplementary material:**

The online version of this article (10.1007/s12471-017-1071-3) contains supplementary material, which is available to authorized users.

## Introduction

**Fig. 1:**
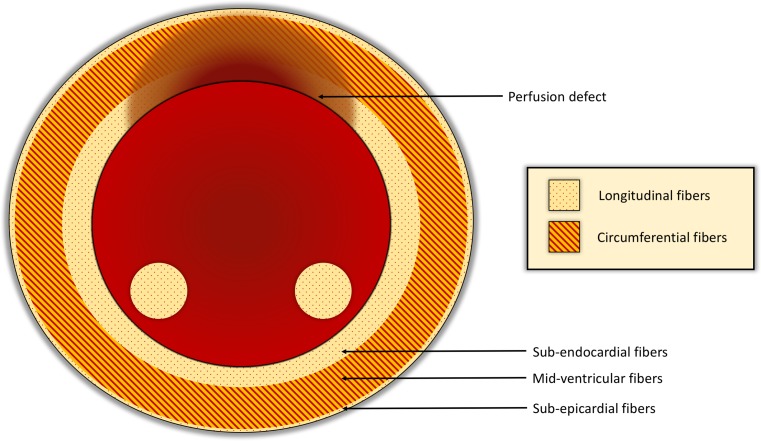
Illustration demonstrating how fibre orientation of the left ventricle corresponds to perfusion defect gradient (high in sub-endocardium and lower in epicardium)

Cardiovascular magnetic resonance (CMR) imaging can detect obstructive coronary artery disease (CAD) by imaging the left ventricular (LV) passage of a contrast bolus during pharmacologically induced myocardial hyperaemia [1]. Although hyperaemic stress does not usually induce myocardial ischaemia per se, myocardium supplied by a significantly stenosed coronary artery shows reduced hyperaemic contrast uptake compared with normal myocardium. Hyperaemia also leads to a redistribution of myocardial blood flow (MBF) between the endocardial and epicardial layers [2]. An endocardial to epicardial gradient of blood flow exists at rest, reflecting the higher metabolic activity of the endocardial layer [3]. In health, pharmacologically induced maximal hyperaemia increases MBF in all myocardial layers although the endocardial to epicardial gradient diminishes as MBF maximises in all myocardial layers. In the context of functionally significant epicardial CAD, hyperaemia leads to a redistribution of MBF from the endocardium to the epicardium, leading to relative endocardial ischaemia, or transmural myocardial steal ([4]; Fig. 1). Thanks to its high in-plane spatial resolution, this transmural perfusion gradient can be demonstrated *in vivo* with first-pass myocardial perfusion CMR and a transmural perfusion gradient of 20% can accurately predict haemodynamically significant CAD as defined by fractional flow reserve (FFR) on invasive coronary angiography [5].

Myocardial strain imaging allows quantification of subtle changes of LV function that typically precede a reduction in LV ejection fraction (EF) [6]. Myocardial deformation can be studied with CMR feature tracking (FT), in which strain is derived from routine cine acquisitions without the need for the previously used tagging methods [7]. FT allows accurate and robust assessment of mainly LV global longitudinal strain [8]. Because longitudinal myocardial fibres are predominantly located in the sub-endocardium, they may be preferentially affected in myocardial ischaemia and by transmural steal during hyperaemia in the presence of significant CAD. The association of differential abnormalities in local left ventricular function assessed by myocardial strain and peak myocardial hyperaemia in the presence or absence of perfusion defects has not been established yet.

Therefore, the purpose of this study was to investigate the relationship between left ventricular global longitudinal strain (GLS), strain rate (GLSR), myocardial early (E’) and late diastolic velocities (A’) with adenosine stress first-pass perfusion CMR and determine which strain parameter is most strongly associated with the presence of a perfusion defect.

## Methods

### Setting

This was a prospective-cohort study of patients presenting to the rapid access chest pain clinic in a single tertiary cardiology centre, who were referred on clinical grounds for a stress CMR study for the evaluation of suspected CAD. Exclusion criteria were: estimated glomerular filtration rate <30 ml/min/1.73 m^2^, non-ischaemic cardiomyopathy or any contraindication to CMR imaging. All patients gave written informed consent for their data to be used in this study.

### Ethics approval

The study protocol was approved by the local research ethics committee. The present study complied with the Declaration of Helsinki and all patients gave written informed consent.

### Image acquisition

CMR protocol included: rest/stress horizontal long-axis (HLA) cine, rest/stress first-pass adenosine perfusion and late gadolinium enhancement imaging. CMR protocol is detailed in the online Supplementary File 1.

### Image analysis

CMR images were anonymised, which included the removal of dates of acquisition and any identifiable data. Cines, perfusion and LGE images were blindly evaluated offline using commercially available software (cvi42 v5.1, Circle Cardiovascular Imaging Inc., Calgary, Canada) by one observer (RA). Left ventricular volumes and ejection fraction (EF) were analysed from short-axis cine images using standard methods [9]. Infarct location was determined by LGE imaging, according to standard guidelines [10].

### Feature tracking strain analysis (rest and stress)

**Fig. 2:**
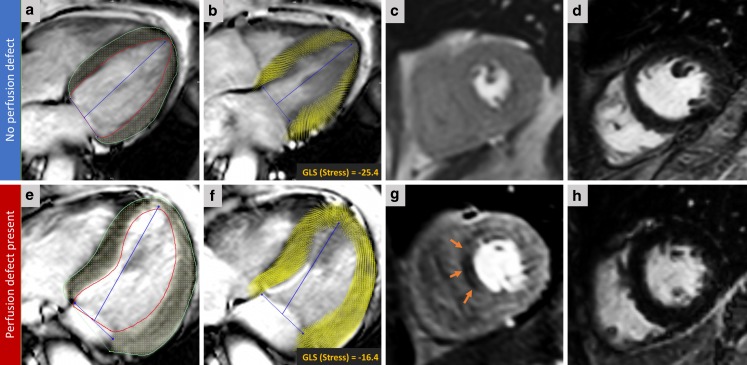
Two case examples: Case 1 (**a–d**). The top row shows CMR images of a 63-year-old female who presented with a history of chest pain. Panel **a** illustrates endo/epi contours on 4‑chamber cine acquisition at peak stress. Panel **b** demonstrates the derived myocardial feature tracking and computed systolic GLS at peak stress. Panel **c** shows the corresponding stress perfusion image at the time of peak myocardial contrast enhancement, showing no inducible perfusion defects. Panel **d** shows that there is no infarction on late gadolinium enhancement (LGE) imaging. Case 2 (**e–h**). The bottom row shows CMR images of a 59-year-old male who presented with a history of chest pain. This is a case with a perfusion defect in mid-ventricular septum (orange arrows in panel **g**) on first-pass perfusion with no evidence of previous myocardial infarction on LGE imaging (Panel **h**). Notably, the peak stress GLS was significantly lower in this case. (*CMR* cardiovascular magnetic resonance, *GLS* global longitudinal strain)

Strain analysis was performed using a cvi42 (v5.1) feature tracking (FT) module in a semi-automated manner (Fig. 2; [11]). FT analysis was done by two observers (GF and PG).

For resting cines, left ventricular endocardial and epicardial borders were manually contoured in end-diastole from both long-axis cines (HLA and VLA). Stress global longitudinal strain parameters were derived from HLA cines only as no VLA images were acquired in order to minimise the duration of adenosine infusion. Peak GLS, GLSR, E’ and A’ were recorded per case.

### Perfusion analysis

Perfusion images were independently analysed by two experts in perfusion analysis with greater than 3‑years’ experience each (TAM and DPR). Each expert reported on the presence of inducible stress perfusion defects that were not present on rest perfusion images and with no corresponding scar on LGE images. In case of disagreement between the two observers, a third independent expert analysed the images, and a discussion of all observers took place to reach a unanimous decision (PG). Studies in which a unanimous decision could not be reached were excluded. On stress perfusion imaging, an area of decreased signal intensity when compared with remote myocardium and the presence of an endocardial to epicardial perfusion gradient were classified as a perfusion defect [12].

### Statistical analysis and sample size estimates

Statistical analysis was performed using IBM SPSS Statistics 21.0. Continuous variables were expressed, as mean ± SD. Normality of quantitative data was established using the Shapiro-Wilk test. Demographic comparisons between two groups of patients (with and without perfusion defect) were performed with an independent samples t‑test. The rest of the statistical methods are detailed in the online Supplementary File 1.

## Results

### Baseline characteristics

A total of 50 patients were recruited; 4 patients had equivocal perfusion results, resulting in exclusion from the study and 2 patients were claustrophobic. From the remaining 44 patients, 22 patients had an inducible perfusion defect, and 22 patients had no inducible perfusion defect. The two independent graders agreed on the categorisation of all cases with no arbitration required. The demographics, clinical data and baseline CMR results are shown in Tab. 1. There were no differences based on gender, age or characteristics present between the groups. Baseline CMR characteristics, including myocardial infarction, were not significantly different in both groups.

**Table 1:** Study demographics and baseline CMR parameters

Characteristics	All patients	Perfusion defect	No perfusion defect	*P*-value
*Demographics*	(*n* = 44)	(*n* = 22)	(*n* = 22)	
Age (years)	64 ± 12	64 ± 12	63 ± 13	0.53
Gender (male/female)	31/13	16/6	15/7	0.75
Current smoker (no. [%])	13 (30)	7 (16)	6 (14)	0.75
Hypertension (no. [%])	13 (30)	7 (16)	6 (14)	0.75
Diabetes Mellitus (no. [%])	12 (27)	6 (14)	7 (16)	0.45
Dyslipidaemia (no. [%])	7 (16)	3 (7)	4 (9)	0.69
Myocardial Infarction (no. [%])	17 (39)	10 (23)	6 (14)	0.22
CABG (no. [%])	6 (14)	4 (9)	1 (2)	0.13
Abnormal ECG (no. [%])	13 (30)	8 (18)	5 (11)	0.33
*Baseline CMR parameters*				
LV EDV, (ml/m^2^)	143 ± 45	151 ± 46	133 ± 43	0.19
LV ESV, (ml/m^2^)	55 ± 32	63 ± 39	45 ± 21	0.06
LV SV, (ml/m^2^)	86 ± 29	87.6 ± 18	84.4 ± 37	0.72
LV EF, (%)	64 ± 13	61 ± 13	67 ± 12	0.07
LV Mass (grams)	111 ± 35	112 ± 26	109 ± 43	0.76
Presence of Infarction (%)	25 (57%)	15 (34%)	10 (23%)	0.13
*Rest strain parameters*				
GLS (%)	−18 ± 4	−16.9 ± 3.7	−19.6 ± 3.4	0.02
GLSR (s^−1^)	−98 ± 11	−86 ± 22	−109 ± 38	0.02
E’ (s^−1^)	80 ± 39	69 ± 31	93 ± 38	0.04
A’ (s^−1^)	80 ± 29	74.5 ± 25	86.7 ± 33	0.18
*Stress strain parameters*				
GLS (%)	−19 ± 4	−16.5 ± 4	−21.2 ± 3.1	<0.001
GLSR (s^−1^)	−104 ± 54	−98 ± 45	−112 ± 60	0.36
E’ (s^−1^)	97 ± 41	90 ± 50	106 ± 32	0.21
A’ (s^−1^)	93 ± 50	88 ± 43	113 ± 81	0.20

Data are presented as mean (standard deviation) or as numbers (%), unless otherwise indicated. *P*-value <0.05 was taken as significant

*A’* myocardial late diastolic velocity, *CABG* coronary artery bypass grafting, *CMR* cardiovascular magnetic resonance, *E’* myocardial early diastolic velocity, *ECG* electrocardiogram, *EDV* end-diastolic volume, *EF* ejection fraction, *ESV* end-systolic volume, *GLS* global longitudinal strain, *GLSR* global longitudinal strain rate, *LV* left ventricular, *SV* stroke volume

### Feature tracking analysis

**Fig. 3:**
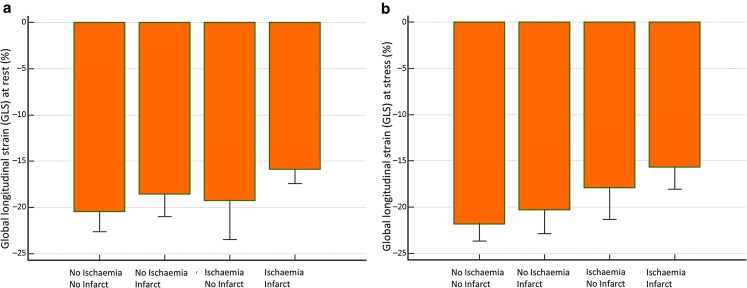
Multiple comparison bars of rest (Panel **a**) and stress (Panel **b**) global longitudinal function strain in patients with/without myocardial infarction and perfusion defect (whiskers: standard deviations; SD)

All cine images were of adequate quality for FT analysis. Fig. 2 demonstrates two cases from the study. Rest GLS, GLSR, E’ and stress GLS were significantly lower in the group with a perfusion defect compared with the no perfusion defect group (Tab. 1). Notably, rest GLS was not significantly different in patients without previous myocardial infarction and with/without ischaemia (Tab. 2; Fig. 3).

**Table 2:** Myocardial deformation parameters in the two patient groups

	Presence of MI	Strain parameters	With perfusion defect	Without perfusion defect	*P*-value
*Rest*	LGE−	GLS (%)	−19 ± 5	−20 ± 3	0.59
		GLSR (s^−1^)	−101 ± 19	−119 ± 53	0.41
		E’ (s^−1^)	94 ± 32	102 ± 52	0.69
		A’ (s^−1^)	93 ± 15	97 ± 36	0.75
	LGE+	GLS (%)	−16 ± 3	−19 ± 4	0.04
		GLSR (s^−1^)	−78 ± 20	−99 ± 13	0.01
		E’ (s^−1^)	56 ± 21	81 ± 30	0.02
		A’ (s^−1^)	66 ± 24	74 ± 25	0.45
*Stress*	LGE−	GLS (%)	−18 ± 4	−22 ± 3	0.02
		GLSR (s^−1^)	−136 ± 55	−102 ± 77	0.32
		E’ (s^−1^)	128 ± 62	111 ± 24	0.39
		A’ (s^−1^)	113 ± 46	113 ± 69	0.99
	LGE+	GLS (%)	−16 ± 4	−20 ± 3	0.01
		GLSR (s^−1^)	−80 ± 27	−125 ± 27	<0.001
		E’ (s^−1^)	72 ± 32	101 ± 40	0.06
		A’ (s^−1^)	76 ± 36	114 ± 97	0.18

Data are presented as mean (standard deviation) or as numbers (%), unless otherwise indicated. *P*-value <0.05 was taken as significant

*A’* myocardial late diastolic velocity, *E’* myocardial early diastolic velocity, *GLS* global longitudinal strain, *GLSR* global longitudinal strain rate, *LGE+* late gadolinium enhancement present, *LGE−* late gadolinium enhancement absent, *MI* myocardial infarction

The absolute change in rest versus stress GLS demonstrated an increase in GLS in patients without perfusion defects but a reduction in GLS at stress in patients with a perfusion defect (−1.6 ± 3.1 versus 0.5 ± 3.8, *p*-value = 0.05). Other strain parameters, GLSR (−2.8 ± 77 versus −12 ± 31, *p*-value = 0.60), E’ (13 ± 45 versus 22 ± 40, *p*-value = 0.45), A (27 ± 65 versus 13 ± 29, *p*-value = 0.43) did not show significant changes between rest and stress.

### Influence of previous myocardial infarction

Patients with previous myocardial infarction on LGE imaging had lower rest GLS (−16 ± 3% vs. −20 ± 4%, *p*-value = 0.007) and stress GLS (−17 ± 4% vs. −20 ± 4%, *p*-value = 0.02). However, patients with previous myocardial infarction did not show more inducible perfusion defects than those without previous myocardial infarction (odds ratio (OR) 0.38, *p*-value = 0.13).

### Receiver operating characteristic curves analysis

**Fig. 4:**
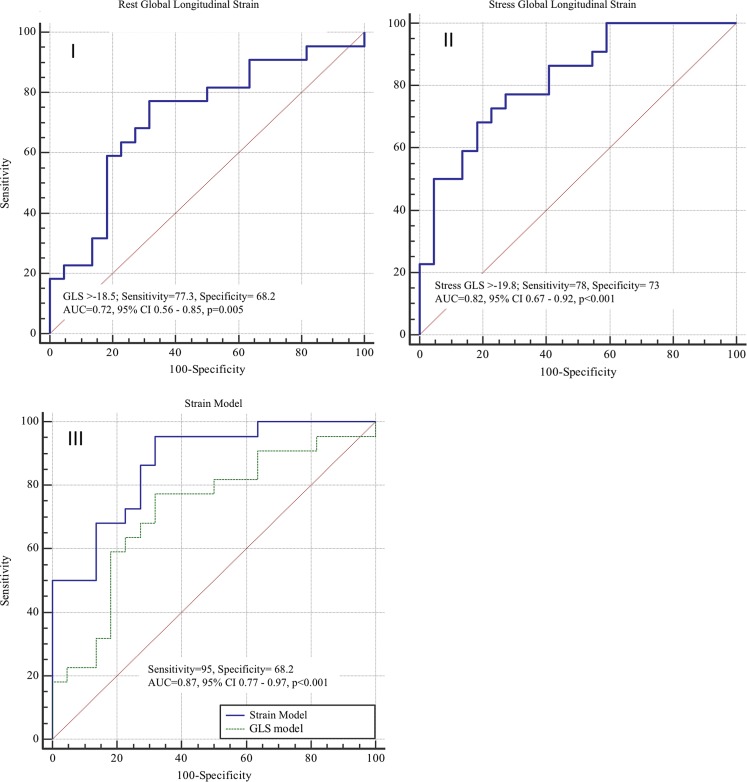
Receiver operating characteristic (ROC) curves of different strain models to predict the presence of myocardial perfusion defect: Resting global longitudinal strain (GLS) model (I), stress GLS (II) and strain model versus rest GLS (III)

Tab. 3 details the diagnostic performance for each of the parameters. Fig. 4 displays the receiver operating characteristic (ROC) plots. Stress GLS displayed a slightly better, though not statistically significant, diagnostic performance compared with rest GLS (Tab. 3; Fig. 4). A strain model comprising of rest GLS, GLSR, E’ and stress GLS demonstrated significant superiority to rest GLS alone. The strain model displayed a sensitivity of 95% and specificity of 68% to detect perfusion defects.

**Table 3:** C-statistics for myocardial longitudinal parameters at rest and stress CMR

		Youden Cut-off	Sensitivity (%)	Specificity (%)	AUC	95% CI	*P*-value
*Rest*	GLS (%)	>−18.55	77.27	68.18	0.72	0.56–0.87	0.006
	GLSR (s^−1^)	>−91.09	68.18	72.73	0.75	0.60–0.89	0.0008
	E’ (s^−1^)	≤84.53	86.36	54.55	0.70	0.54–0.86	0.01
	A’ (s^−1^)	≤108.86	100	32	0.59	0.42–0.77	0.28
*Stress*	GLS (%)	>−19.80	77.3	72.7	0.82	0.70–0.94	<0.001
	GLSR (s^−1^)	>−99.7	63.6	86.4	0.74	0.58–0.89	0.003
	E’ (s^−1^)	≤81.65	50	82	0.67	0.50–0.83	0.04
	A’ (s^−1^)	≤58.65	36.36	86.36	0.58	0.41–0.76	0.34
*Strain model* ^a^		28%	96	68	0.87	0.76–0.97	<0.0001

Data as presented as mean (standard deviation) or as numbers (%), unless otherwise indicated. *P*-value <0.05 was taken as significant

*A’* myocardial late diastolic velocity, *AUC* area under the curve, *CI* confidence interval, *CMR* cardiovascular magnetic resonance, *E’* myocardial early diastolic velocity, *GLS* global longitudinal strain, *GLSR* global longitudinal strain rate

^a^Model comprising of strain parameters associated to the presence of perfusion defect in univariate analysis: rest GLS, rest GLSR, rest E’ and stress GLS

### Regression analysis

In the logistic regression analysis, stress GLS demonstrated the best independent association with the presence of a perfusion defect of the parameters tested (OR 1.43 95% CI 1.14–1.78, *p*-value <0.001) (Online Supplementary File 2). The logistic regression strain model was independently associated with presence of perfusion defect (*p*-value <0.001) when compared with other individual myocardial strain parameters.

## Discussion

The main novel findings of this study are: 1) at peak myocardial hyperaemia, GLS is reduced in patients with inducible perfusion defects; 2) stress GLS is most strongly associated with the presence of a perfusion defect; and 3) a cut-off value of −19.8% for stress GLS demonstrates 77% sensitivity and 73% specificity for the presence of a perfusion defect.

Myocardial ischaemia initially affects the endocardium and progresses to the sub-epicardial layers in a ‘wave front’ manner [13]. High resolution adenosine stress myocardial perfusion CMR can demonstrate a transmural gradient of myocardial perfusion in patients with flow limiting CAD, representing the redistribution of myocardial blood flow from the sub-endocardium to the sub-epicardium. Sub-endocardial fibres are structurally longitudinal fibres [14] and therefore predominantly contribute to the longitudinal function of the left ventricle [15]. The main findings of the present study are consistent with these known concepts. We found that global longitudinal function assessed by GLS was adversely affected during adenosine stress in patients with perfusion defects while GLS in patients with no perfusion defects increased during hyperaemia. The most likely mechanism underpinning this observation is that relative ischaemia of the sub-endocardial myocardial layer (‘transmural myocardial steal’) affects longitudinal fibre function during hyperaemia and thus differentially reduces longitudinal LV function.

In patients with evidence of myocardial infarction on LGE imaging, rest GLS, GLSR and E’ were also correlated with the presence of perfusion defects, however, these resting strain parameters did not discriminate between patients with and without perfusion defects in the absence of previous myocardial infarction. Like ischaemia, myocardial infarction predominantly affects the endocardial layer and a longitudinal myocardial strain and a reduction in resting myocardial deformation can therefore be expected. The correlation with the presence of an inducible perfusion defect is likely to be caused by co-existing CAD in other territories or peri-infarct ischaemia, both of which were common in the present population in patients with prior MI. However, resting strain parameters are not reliable markers of inducible ischaemia as shown by the lack of correlation with perfusion defects in patients without myocardial infarction.

A strain model comprising of rest GLS, GLSR, E’ and stress GLS performed slightly better in this study than stress GLS alone in linear regression (Tab. 3), but was not statistically superior to individual parameters in area under the curve (AUC) analysis (*p* > 0.05). As the strain model requires multiple strain analyses, the use of stress GLS alone may be a more practical approach for clinical studies.

Previous echocardiographic studies have reported findings that are consistent with our observations. Liang et al. found that rest peak systolic strain rate (equivalent to GLSR in our study) and peak early diastolic strain rate (E’ in our study) were significantly lower in patients with significant CAD (>70% stenosis) than controls [16]. Our study demonstrated similar global resting strain rate to Liang et al. (Tab. 1). However, our study was able to accurately differentiate patients with previous myocardial infarction on LGE imaging and demonstrate clear differences of strain rate at rest in patients with/without previous myocardial infarction (Tab. 2). A pre-clinical porcine study by Reant et al. also demonstrated that flow reduction in the coronary artery achieved by adenosine-induced myocardial hyperaemia (flow reduction by 70%) adversely affected myocardial deformation parameters (mainly longitudinal and circumferential strain) at stress [17]. In a multi-centre study of 102 patients who underwent concomitant dobutamine stress echocardiography and coronary angiography, longitudinal strain at peak stress demonstrated better diagnostic accuracy than wall motion score [18]. In the same study, a dobutamine stress GLS cut-off of −20% demonstrated 84% sensitivity and 87% specificity for significant CAD. The optimum cut-off for stress GLS in our study was very similar at −19.8%.

## Study limitations

The sample size of this proof-of-concept study is small, although large enough to detect statistically significant differences on logistic regression analysis and thus justifying larger studies to investigate this concept further. For practical and conceptual reasons, we did not use coronary stenosis on invasive angiography but perfusion defects on myocardial perfusion CMR as the primary end-point [19]. Contemporary CMR pulse-sequences for first-pass perfusion are highly accurate for the diagnosis of significant ischaemia [20]. This work is hypothesis-generating research and offers mechanistic insights which need to be validated against the gold standard for physiologically significant ischaemia, invasive FFR. Our results may not be applicable to patients with infiltrative cardiomyopathies (hypertrophic cardiomyopathy, cardiac amyloidosis, sarcoidosis etc.), where stiffening of the left ventricle may affect myocardial deformation [21]. Several papers have demonstrated that the aforementioned infiltrative cardiomyopathies lead to reduced GLS so that adenosine stress GLS analysis may not be reliable [22, 23]. Importantly, this study also had a few technical limitations. Stress myocardial deformation was only assessed in one plane, i. e. the 4‑chamber cine. Strain rate imaging parameters derived by FT suffer from low temporal resolution. Even though FT-derived strain analysis is very reliable for global assessment, its reliability at regional level assessment is debatable [8], mainly because of intra-/inter-observer variability. Hence, this was not done in the present study.

## Conclusion

In this mechanistic study, at peak myocardial hyperaemic stress, GLS is reduced in the presence of a myocardial perfusion defect, most likely secondary to reduced endocardial blood flow as a result of hyperaemia-induced redistribution of transmural perfusion. Additionally, this study demonstrates the feasibility of adenosine stress myocardial strain CMR which may provide clinically relevant information and justifies further larger studies to investigate the accuracy of using CMR-FT-derived strain to predict the presence of CAD.

## Caption Electronic Supplementary Material

Detail description of CMR protocol and statistical methods

Regression analysis table
