# Supplementary material for: Effects of hyperaemia on left ventricular longitudinal strain in patients with suspected coronary artery disease: A first-pass stress perfusion cardiovascular magnetic resonance imaging study
Source: Neth Heart J. 2018 Jan 8;26(2):85–93. doi: 10.1007/s12471-017-1071-3 (PMC5783894; doi:10.1007/s12471-017-1071-3)
Supplement: Supplementary file 1 — Detail description of CMR protocol and statistical methods [file 12471_2017_1071_MOESM1_ESM.docx]

**Supplementary File 1**

**Image acquisition**

All patients had CMR imaging on a 1.5T MR system (Ingenia, Philips Healthcare, Best, The Netherlands) equipped with dStream technology. A dedicated 28-channel cardiac phased array receiver coil was used. CMR imaging was performed using standard protocols [1].

**CMR Protocol**

**CMR protocol is detailed below (S Fig 1):**

1. Free breathing low-resolution survey scan of the chest

2. Long-axis cines: Vertical long axis (VLA), horizontal long axis (HLA) acquired with a balanced steady-state free precession (bSSFP), single-slice breath-hold sequence. Typical parameters for bSSFP cine were as follows: echo time 1.3 milliseconds, repetition time 2.6 milliseconds, flip angle 40°, field of view 320-420 mm according to patient size, sensitivity encoding (SENSE) acceleration (undersampling factor 2), slice thickness 10 mm, and 30 phases per cardiac cycle.

3. Stress perfusion imaging: To achieve maximal hyperaemia, adenosine was administered by continuous intravenous infusion at a dose of 140 μg kg−1 min−1 for at least 4 minutes. Adequate hemodynamic response was assessed by either ≥10% heart rate increase or ≥10 mm Hg decrease in systolic blood pressure. If there was an inadequate hemodynamic response, the dose was increased incrementally to 170 μg kg−1 min−1 and then 210 μg kg−1 min−1 for a further 2 minutes until hemodynamic response was achieved. Perfusion image acquisition used a 2-dimensional, T1-weighted saturation recovery–prepared gradient echo-pulse sequence in 3 short-axis slices, planned using the 3/5 technique, using SENSE (undersampling factor 2) [2]. A bolus intravenous injection of 0.05 mmol/kg dimeglumine gadopentetate (Magnevist®, Schering AG, West Sussex, UK) followed by a 15 ml saline flush was delivered through an arm vein at 5 ml/s using a power injector (Spectris®, Medrad, Pittsburgh, Pennsylvania), during breath hold in end-expiration.

4. Stress HLA: First-pass perfusion imaging was immediately followed by the acquisition of a repeat HLA cine (all parameters similar to the resting pre-stress HLA) while adenosine was still being infused.

5. Short-axis LV cine stack: Resting wall motion and left ventricular function were assessed with a contiguous stack of multiphase ventricular short-axis balanced steady-state free precession cines (10-12 slices, 30 phases, 10-mm slice thickness, 0-mm gap, same cine pulse sequence as above).

6. Rest perfusion imaging: Rest perfusion images were acquired using an identical pulse sequence as stress images, with the same slice positioning and injection characteristics to the stress perfusion scan and was performed after waiting for 15 minutes.

7. Scar imaging: Late gadolinium enhancement (LGE) was performed in 10 to 12 short-axis and orthogonal HLA and VLA orientations from 10 minutes after rest perfusion with an inversion recovery–prepared T1-weighted gradient echo-pulse sequence. Typical parameters are as follows: echo time 2.0 milliseconds, repetition time 3.7 milliseconds, flip angle 25°, acquired spatial resolution 0.70 × 0.70 × 10 mm3, and inversion time individually adjusted per inversion time scout.

**
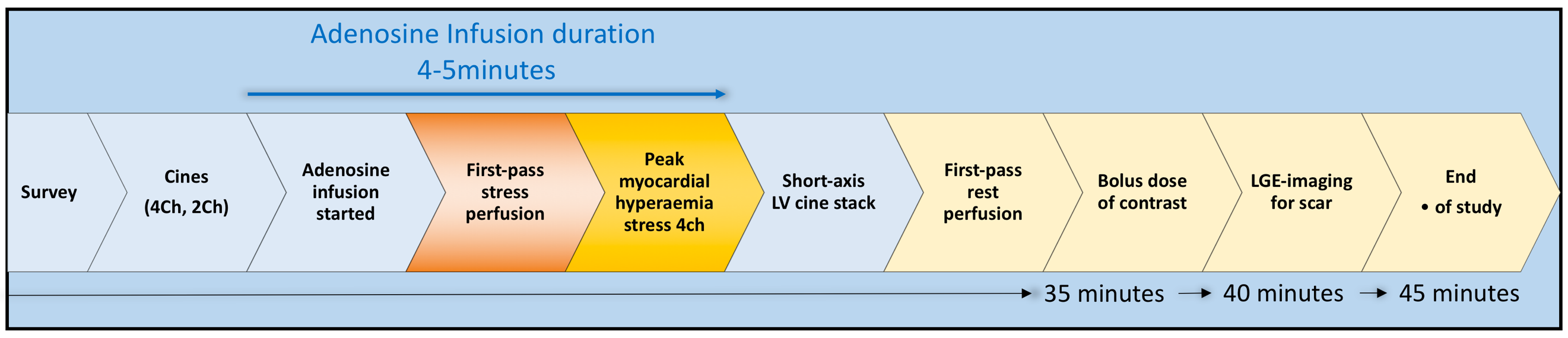
S Fig. 1.** CMR protocol and time-line of different acquisitions.

**Statistical methods and power calculation**

A repeated-measures analysis of variance (ANOVA) was performed on patient demographics and CMR parameters in the two main groups depending on the presence of perfusion defect. The accuracy of myocardial deformation parameters in predicting the presence of a perfusion defect was examined using the receiver-operator characteristic (ROC) curve analyses described by Delong for comparison of the area under the curve (AUC), using Medcalc (v15.8). Forward (conditional) multivariable logistic regression was used for all strain parameters with statistical significance from one-way analysis (p<0.1). A linear regression model, comprising of the predictive values was generated for all the strain parameters which demonstrated significant association to the presence of perfusion defect in univariate analysis. All statistical tests were 2-tailed; P-values <0.05 were considered significant. For sample size estimation, we assumed to that there will be a mean difference of 3% between patient’s with/without perfusion defect in their stress GLS. Informed from our previous work, we took 3% standard deviation for both groups [3]. As per Machin et at’s described methods, we needed to recruit at-least 17 in each group to achieve a power of 80% and with an alpha of 5% [4].

**Reference:**

1. Schulz-Menger J, Bluemke DA, Bremerich J, Flamm SDS, Fogel MA, Friedrich MGM, et al. Standardized image interpretation and post processing in cardiovascular magnetic resonance: Society for Cardiovascular Magnetic Resonance (SCMR) board of trustees task force on standardized post processing. J. Cardiovasc. Magn. Reson. [Internet]. BioMed Central; 2013 [cited 2014 Nov 20];15:35. Available from: http://www.pubmedcentral.nih.gov/articlerender.fcgi?artid=3695769&tool=pmcentrez&rendertype=abstract

2. Messroghli DR, Bainbridge GJ, Alfakih K, Jones TR, Plein S, Ridgway JP, et al. Assessment of regional left ventricular function: accuracy and reproducibility of positioning standard short-axis sections in cardiac MR imaging. Radiology [Internet]. 2005 [cited 2015 Aug 12];235:229–36. Available from: http://www.ncbi.nlm.nih.gov/pubmed/15731374

3. Fent GJ, Garg P, Foley JRJ, Dobson LE, Musa TA, Erhayiem B, et al. The utility of global longitudinal strain in the identification of prior myocardial infarction in patients with preserved left ventricular ejection fraction. Int. J. Cardiovasc. Imaging [Internet]. Springer Netherlands; 2017 [cited 2017 May 17];1–9. Available from: http://link.springer.com/10.1007/s10554-017-1138-7

4. Machin D, Campbell M, Fayers, P PA. Sample Size Tables for Clinical Studies. Second Ed. Blackwell Sci. IBSN. 1997;18–20.
