# Supplementary material for: Effects of hyperaemia on left ventricular longitudinal strain in patients with suspected coronary artery disease: A first-pass stress perfusion cardiovascular magnetic resonance imaging study
Source: Neth Heart J. 2018 Jan 8;26(2):85–93. doi: 10.1007/s12471-017-1071-3 (PMC5783894; doi:10.1007/s12471-017-1071-3)
Supplement: Supplementary file 2 — Regression analysis table [file 12471_2017_1071_MOESM2_ESM.docx]

**Supplementary File 2**

**Table.** Predictors of perfusion defect in univariable (UV) and multivariable (MV) logistic regression analysis.

|  | Variable | UV | | | MV (without model) | MV (with model) |
| --- | --- | --- | --- | --- | --- | --- |
|  |  | OR | 95% CI | P-value | P-value | P-value |
| Rest | GLS | 1.25 | 1.03 - 1.52 | 0.02 | 0.74 | 0.86 |
|  | GLSR | 1.04 | 1.01 - 1.07 | 0.02 | 0.06 | 0.77 |
|  | E’ | 0.98 | 0.96 - 1.00 | 0.05 | 0.32 | 0.72 |
|  | A’ | 0.99 | 0.96 - 1.01 | 0.18 | … | … |
| Stress | GLS | 1.43 | 1.14 - 1.78 | 0.002 | <0.001 | 0.68 |
|  | GLSR | 1.01 | 0.99 - 1.02 | 0.37 | … | … |
|  | E’ | 0.99 | 0.97 - 1.01 | 0.21 | … | … |
|  | A’ | 0.99 | 0.98 - 1.00 | 0.22 | … | … |
| Strain Model^@^ | 28% |  |  | <0.001 | … | <0.0001 |

P-value <0.05 was taken as significant. CI, confidence interval; OR, odds ratio; UV, univariable; MV,

^@^ Model comprising of strain parameters associated to the presence of perfusion defect in univariate analysis: rest GLS, rest GLSR, rest E’ and stress GLS
